# Immune system disruptions implicated in whole blood epigenome-wide association study of depression among Parkinson's disease patients

**DOI:** 10.1016/j.bbih.2022.100530

**Published:** 2022-10-03

**Authors:** Kimberly C. Paul, Cynthia Kusters, Melissa Furlong, Keren Zhang, Yu Yu, Aline Duarte Folle, Irish Del Rosario, Adrienne Keener, Jeff Bronstein, Janet S. Sinsheimer, Steve Horvath, Beate Ritz

**Affiliations:** a Department of Neurology, David Geffen School of Medicine, Los Angeles, CA, USA; b Departments of Human Genetics, David Geffen School of Medicine, Los Angeles, CA, USA; c University of Arizona, Mel and Enid Zuckerman College of Public Health, Tucson, AZ, USA; d Department of Epidemiology, UCLA Fielding School of Public Health, Los Angeles, CA, USA; e Center for Health Policy Research, UCLA Fielding School of Public Health, Los Angeles, CA, USA; f Department of Biostatistics, UCLA Fielding School of Public Health, Los Angeles, CA, USA

**Keywords:** Parkinson's disease, Depression, DNA methylation, Inflammation, Methylation QTLs, Neutrophil-to-lymphocyte ratio

## Abstract

Although Parkinson's Disease (PD) is typically described in terms of motor symptoms, depression is a common feature. We explored whether depression influences blood-based genome-wide DNA methylation (DNAm) in 692 subjects from a population-based PD case-control study, using both a history of clinically diagnosed depression and current depressive symptoms measured by the geriatric depression scale (GDS). While PD patients in general had more immune activation and more accelerated epigenetic immune system aging than controls, the patients experiencing current depressive symptoms (GDS≥5) showed even higher levels of both markers than patients without current depressive symptoms (GDS<5). For PD patients with a history of clinical depression compared to those without, we found no differences in immune cell composition. However, a history of clinical depression among patients was associated with differentially methylated CpGs. Epigenome-wide association analysis (EWAS) revealed 35 CpGs associated at an FDR≤0.05 (569 CpGs at FDR≤0.10, 1718 CpGs at FDR≤0.15). Gene set enrichment analysis implicated immune system pathways, including immunoregulatory interactions between lymphoid and non-lymphoid cells (p-adj = 0.003) and cytokine-cytokine receptor interaction (p-adj = 0.004). Based on functional genomics, 25 (71%) of the FDR≤0.05 CpGs were associated with genetic variation at 45 different methylation quantitative trait loci (meQTL). Twenty-six of the meQTLs were also expression QTLs (eQTLs) associated with the abundance of 53 transcripts in blood and 22 transcripts in brain (substantia nigra, putamen basal ganglia, or frontal cortex). Notably, cg15199181 was strongly related to rs823114 (SNP-CpG p-value = 3.27E-310), a SNP identified in a PD meta-GWAS and related to differential expression of *PM20D1*, *RAB29*, *SLC41A1*, and *NUCKS1*. The entire set of genes detected through functional genomics was most strongly overrepresented for interferon-gamma-mediated signaling pathway (enrichment ratio = 18.8, FDR = 4.4e-03) and T cell receptor signaling pathway (enrichment ratio = 13.2, FDR = 4.4e-03). Overall, the current study provides evidence of immune system involvement in depression among Parkinson's patients.

## Highlights

• Parkinson's disease (PD) is associated with clinical depression prior to PD onset and depressive symptoms after PD diagnosis.
• Epigenome-wide analysis revealed CpGs related to current depressive symptoms and a history of clinical depression among PD patients.
• Patients experiencing current depressive symptoms had the highest epigenetic-based neutrophil-to-lymphocyte ratio on average.
• Patients with a history of clinical depression had differentially methylated CpGs in genes enriched for immune system pathways.
• Many of the depression associated CpGs were linked to differential expression through meQTL/eQTLs, which included GWAS variants.

## 1 Introduction

Parkinson's disease (PD) is a progressive neurodegenerative disorder characterized by gradual loss of dopaminergic neurons in the substantia nigra region of the brain. While PD is typically described in terms of motor symptoms, neuropsychiatric symptoms and especially depression are common, both preceding and subsequent to motor symptom onset (Aarsland et al., 2012). Depression in PD is strongly related to diminished health-related quality of life, reduced function, and cognitive decline (Aarsland et al., 2012; Lieberman, 2006). Although often underdiagnosed, clinically relevant depressive symptoms occur in an estimated 35% of PD patients and epidemiologic evidence indicates a strong association between a history of major depression and subsequent development of PD(1).

The question remains whether depression is a prodromal symptom of PD or a risk factor for PD incidence. However, whichever is the case, there is mounting evidence for biologic underpinnings of depression in PD, including structural changes in the brain of depressed PD patients relative to non-depressed patients (e.g. loss of striatal dopamine transporter availability (Weintraub et al., 2005); loss of white matter within the cortico–limbic network (Kostić et al., 2010)), alterations in neurotransmitter systems (e.g. dopaminergic and serotonergic circuits (Grosch et al., 2016; Lu et al., 2019; Mayberg et al., 1990)), and an involvement of inflammatory and neurotrophic factors (e.g. extended stress-induced activation of the brain via cytokines and glucocorticoids (Pace and Miller, 2009); downregulation of the MAPK–MEK pathway (Wang and Mao, 2019)).

Depression likely arises due to a combination of environmental and genetic factors (Aarsland et al., 2012). Currently, we have a limited understanding of etiologic mechanisms that contribute to depression in PD. Here we are concentrating on DNA methylation (DNAm) as it may offer a readout for biologic pathways that are impacted. DNAm also regulates gene expression/repression (Moore et al., 2013) and it also reflects both environmental and genetic determinants (Law and Holland, 2019). Specifically, DNAm alterations may reflect key mechanisms through which exposures interact with genetic predisposition to determine an individual's susceptibility. Furthermore, depression has also long been linked to immune dysregulation (Maes, 1995). For instance, PD, depression, and related risk factors, such as psychosocial stressors, can induce proinflammatory cytokines and have been associated with sustained epigenetic changes (Slavich and Irwin, 2014; Reale et al., 2009; Cunliffe, 2016; Zannas, 2019). Thus, blood may be an informative tissue for assessing depression related DNAm changes.

To investigate blood-based DNA methylation related to depression in PD, we performed an epigenome-wide association study (EWAS) of both having a history of clinical depression and current depressive symptoms in 692 participants of a population-based case control study of PD with longitudinal data for patients. We further used publicly available data (GTEx and BIOS) to map the associated CpGs to different biologic layers, using methylation and expression quantitative trait loci (QTL) to assess networks of CpGs, SNPs, and transcripts related to depression in PD.

## 2 Results

Our analysis of blood-based DNAm draws from participants recruited as part of the Parkinson's disease, Environment, and Genes (PEG) study (Ritz et al., 2016). Data are available on Gene Expression Omnibus (GEO), accession numbers GSE72774 and GSE72776(18,19). PEG is a population-based study of Parkinson's disease, which enrolled patients and controls from California's Central Valley (2001–2007 & 2010–2016). Primary analysis was restricted to 465 PD patients of European ancestry (based on AIMs ancestry markers) for whom we had DNAm data available and 227 controls of European ancestry. Given the limited sample size, we used the control population only to assess whether depression associations seen among the PD patients were also seen among the controls (i.e., related to depression in general, or related to depression in PD specifically). Characteristics of the study population can be found in Table 1.

**Table 1:** Study characteristics (n = 692).

	PD Patients (n = 465)				Controls (n = 227)		
	No Depression	Depression History	Lower Depressive Symptoms (GDS 1–4)	Higher Depressive Symptoms (GDS 5+)	No Depression or Depressive Symptoms	Depression History	Higher Depressive Symptoms (GDS 5+)
n (%) or Mean (SD)	n = 335	n = 130	n = 334	n = 130	n = 167	n = 49	n = 23
Age at blood draw	71.8 (9.4)	68.8 (10.0)*	71.2 (9.43)	70.2 (10.3)	69.4 (12.0)	62.1 (13.0)*	62.1 (13.0)
Age at diagnosis	69.4 (9.5)	66.0 (10.1)*	68.9 (9.5)	67.3 (10.4)	–	–	–
Male	211 (63)	71 (55)	195 (58)	86 (66)	102 (61)	35 (51)*	11 (48)
PD duration at baseline	2.6 (2.0)	3.1 (2.5)*	2.5 (2.0)	3.4 (2.5)*	–	–	–
Ever Smoker	148 (44)	67 (52)	150 (45)	65 (50)	95 (57)	29 (59)	20 (87)*
**Baseline Motor Scores**							
HY3+ (yes)	58 (18)	23 (19)	45 (14)	35 (28)	–	–	–
UPDRS-III	21.0 (10.8)	23.2 (12.0)	19.2 (9.3)	27.9 (13.0)*	–	–	–
Bradykinesia	1.1 (0.9)	1.3 (0.9)	1.0 (0.8)	1.5 (1.0)*	–	–	–
Rigidity	3.4 (2.3)	3.9 (2.6)	3.2 (2.1)	4.4 (2.9)*	–	–	–
Tremor	3.1 (2.6)	3.1 (2.8)	2.9 (2.4)	3.6 (3.2)*	–	–	–
Gait/Balance	1.6 (1.4)	2.0 (1.9)*	1.4 (1.3)	3.4 (1.9)*	–	–	–
Axial Score	4.3 (3.1)	4.9 (3.5)	3.9 (2.7)	6.1 (3.8)*	–	–	–
Levodopa Use (yes)	243 (73)	92 (72)	237 (71)	97 (76)	–	–	–
LED (mg/day)	300 (268)	336 (294)	292 (271)	356 (282)*	–	–	–

*p < 0.05.

We assessed two indicators related to depression, first, having a history of clinical depression based on self-reported physician diagnosis and, second, having current depressive symptoms measured by the geriatric depression scale (GDS) at the time of blood draw. We dichotomized the GDS into groups with no or low depressive symptoms (GDS<5) and with depressive symptoms (GDS≥5). We selected this GDS cut-point as we have previously validated the indicator (GDS≥5) as having high specificity and sensitivity in distinguishing minor and major depression in a subset of our patient population (Thompson et al., 2011). This validity has also been shown in other studies of PD(21). An overview of the analysis plan is shown in Supplemental Fig. 1.

Overall, 130 PD patients (28%) reported a history of clinical depression and 130 PD patients (28%) had current depressive symptoms at baseline. The two depression indicators were moderately correlated among the PD patients (Pearson's R = 0.32, 95% CI = 0.25, 0.39), with 70 of the patients having both a history of clinical depression and experiencing current depressive symptoms at the time of blood draw.

### 2.1 Immune cell composition and current depression

**Fig. 1:**
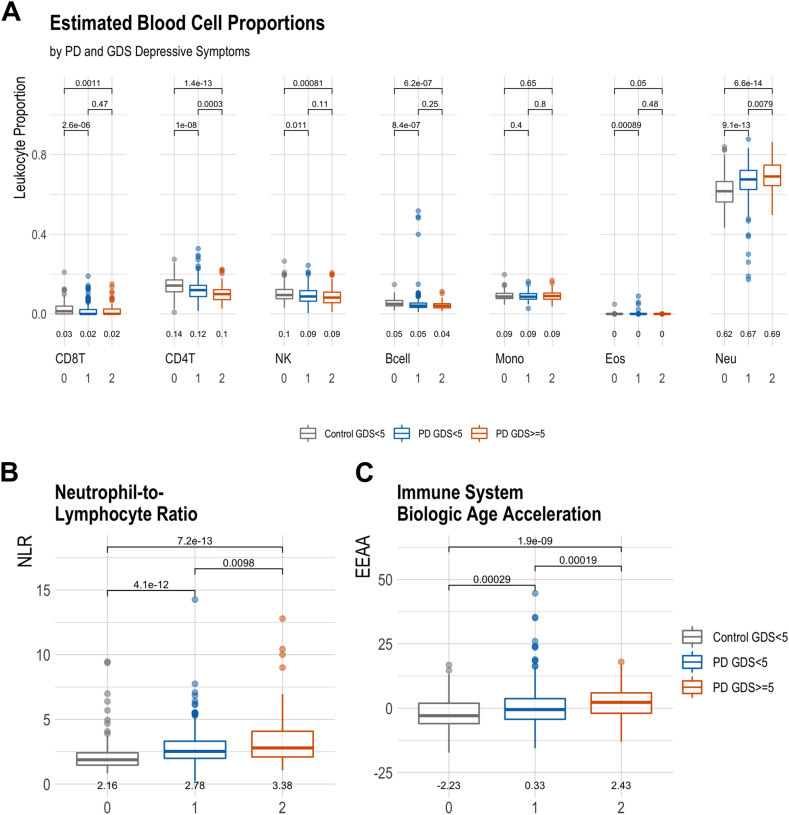
**Epigenetic Immune System Markers and Current Depressive Symptoms in PD.** Mean comparisons of **A)** Houseman DNAm estimated leukocyte proportions, **B)** Neutrophil-to-lymphocyte ratio (based on DNAm leukocyte proportions), and **C)** epigenetic immune system age acceleration (EEAA) across three groups: controls (no depressive symptoms), PD patients without depressive symptoms (GDS<5), and PD patients with depressive symptoms (GDS>5).

Using methylation levels as a surrogate for leukocyte composition (Houseman method (Houseman et al., 2012; Houseman et al., 2014)), we observed that current depressive symptoms were strongly related to differential levels of several epigenetic immune markers (Fig. 1). PD patients with current depressive symptoms (GDS≥5) on average had the highest proportion of neutrophils (69%, SD = 7%; Fig. 1A), followed by PD patients without depressive symptoms (67%, SD = 9%; Wilcoxon p = 0.008 comparing PD patients with and without depressive symptoms), and controls without depressive symptoms (61%, SD = 8%; p = 6.1e-14 comparing PD patients with depressive symptoms to controls). The controls without depressive symptoms had the highest proportion of CD4T cells (15%, SD = 5%), followed by PD patients without depressive symptoms (12%, SD = 5%), and PD patients with depressive symptoms (10%, SD = 4%). Similar trends were also seen for other lymphocytes, notably CD8T, NK, and B cells. When combining cell composition markers into a neutrophil-to-lymphocyte (NLR) ratio, the PD patients with depressive symptoms had a significantly higher ratio (mean = 3.38, SD = 1.9) than both PD patients without depressive symptoms (mean = 2.78, SD = 1.4; p = 0.0098) and the controls (mean = 2.16, SD = 1.2; p7.2e-13; Fig. 1B).

Given the differences we observed in immune cell composition, we assessed immune system epigenetic age acceleration as measured by the extrinsic epigenetic aging clock (Horvath and Levine, 2015). PD patients with depressive symptoms had significantly more immune system age acceleration (mean = 2.43 years, SD = 6.1) than both controls (mean = −2.23, SD = 6.2; p = 1.9e-9) and patients without depressive symptoms (mean = 0.33, SD = 7.5; p = 1.9e-4; Fig. 1C).

However, when examining patients by a history of clinical depression instead of based on current depressive symptoms (GDS≥5), we found these differences were limited to current symptoms. Immune cell composition was similar among PD patients with and without a history of clinical depression (Supplemental Fig. 2). Furthermore, the observed differences were also specific to PD patients. Non-PD controls with higher levels of depressive symptoms (GDS≥5) and a history of clinical depression had a similar cell composition and immune system age acceleration as controls without depressive symptoms or a history of clinical depression (Supplemental Fig. 3).

### 2.2 Depression epigenome-wide association study

In our primary analysis, we related the two depression indicators to genome-wide DNAm levels among PD patients, adjusting for cell composition (proportion of neutrophils and CD4Ts), age, sex, smoking, PD duration at baseline, European fractional ancestry, and study wave. Without controlling for cell composition, the GDS≥5 indicator for current depressive symptoms was related to 129 CpGs at an FDR<0.05 and 675 CpGs at an FDR<0.10 among PD patients. However, after controlling for cell composition, the current depressive symptoms indicator was not related to any CpG levels in site-by-site analysis (p > 7.1e-6; FDR>0.99).

On the other hand, having a history of clinical depression prior to PD onset was associated with several differentially methylated positions (DMP). An overview of the results is displayed in a Manhattan plot (Supplemental Fig. 4). Overall, having a history of clinical depression was associated with 35 CpGs at an FDR<0.05, 569 CpGs at an FDR<0.10, 1718 CpGs at an FDR<0.15, and 5270 CpGs at an FDR<0.25 (Supplemental Table 1). The top CpGs based on FDR are detailed in Table 2. The most significantly associated CpG was cg18774195 (FDR = 0.007) in the 5′UTR region of the *SLC7A14* gene. Other notable CpGs included cg23426156 and cg11042505, linked to schizophrenia in EWAS(25,26), 32 CpGs linked to aging, and 6 CpGs linked to smoking. These CpGs are listed in the supplemental materials and were determined via query of the MRC-IEU catalog of epigenome-wide association studies (Battram et al., 2022). In sensitivity analysis, we assessed whether the CpGs associated with clinical depression among PD patients were also associated with a history of clinical depression among controls (Supplemental Table 1). From the 35 CpGs associated at FDR<0.05 among the PD patients, three CpGs, cg22801913, cg21276379, and cg25290938 were also associated with depression among controls at p < 0.05. The three CpGs were located in body of gene *C11orf49*, the transcription start site of *EPAS1*, and the transcription start site of *ARHGAP22*, respectively, and all three were also associated with aging in a prior EWAS(27). Otherwise, of the 5270 CpGs associated at FDR<0.25 among the patients, 242 in total were also associated with depression among controls (p < 0.05), indicating that among our population most of the depression CpGs were specific to depression among PD.

**Table 2:** EWAS top hits: Associating a history of clinical depression to genome-wide methylation levels among PD patients with depression relative to PD patients without depression.

CpG	chr	UCSC RefGene Name	P-value	FDR	β	95% CI	Relation to Island	UCSC RefGene Group	DMR	Enhancer	DHS
cg18774195	chr3	SLC7A14	2.07E-08	0.007	0.020	0.03, 0.01	S_Shore	5′UTR; 1stExon			
cg26297819	chr22	TEF	1.14E-07	0.012	0.021	0.03, 0.01	OpenSea	TSS200		TRUE	
cg09047573	chr5	NME5	1.18E-07	0.012	0.033	0.05, 0.02	OpenSea	5′UTR			TRUE
cg16646909	chr19	ZNF790	1.36E-07	0.012	0.009	0.01, 0.01	Island	TSS1500	DMR		TRUE
cg01949993	chr1	LAMC2	1.71E-07	0.012	0.026	0.04, 0.02	OpenSea	TSS200			TRUE
cg21769117	chr6	CLIC1	2.12E-07	0.012	0.021	0.03, 0.01	N_Shelf	TSS1500			
cg20476159	chr18	CTDP1	2.71E-07	0.014	0.022	0.03, 0.01	N_Shelf	Body			
cg13209762	chr1		4.17E-07	0.016	0.020	0.03, 0.01	OpenSea			TRUE	
cg24092282	chr14		4.27E-07	0.016	0.032	0.04, 0.02	Island			TRUE	
cg06040872	chr17	CCL18	4.75E-07	0.016	−0.022	−0.01, −0.03	OpenSea	Body			
cg09901574	chr16		5.18E-07	0.016	−0.006	0.00, −0.01	N_Shelf				
cg09554876	chr1	BEND5; AGBL4	5.61E-07	0.016	−0.012	−0.01, −0.02	OpenSea	Body			
cg23426156	chr8		6.13E-07	0.017	−0.028	−0.02, −0.04	OpenSea			TRUE	
cg21811896	chr1	MEGF6	6.81E-07	0.017	−0.012	−0.01, −0.02	OpenSea	Body			
cg22668767	chr5		7.32E-07	0.017	0.031	0.04, 0.02	S_Shore		RDMR		
cg15199181	chr1		7.92E-07	0.017	0.024	0.03, 0.01	OpenSea			TRUE	
cg06586775	chr20	SPAG4	8.01E-07	0.017	0.006	0.01, 0.00	Island	Body			
cg05124308	chr1	TMEM200B	1.24E-06	0.024	0.010	0.01, 0.01	Island	TSS200			
cg05868564	chr11		1.38E-06	0.026	0.021	0.03, 0.01	OpenSea			TRUE	
cg22801913	chr11	C11orf49	1.65E-06	0.029	0.010	0.01, 0.01	OpenSea	Body		TRUE	
cg24389488	chr17	WNT3	1.74E-06	0.029	0.015	0.02, 0.01	Island	Body			
cg21276379	chr2	EPAS1	2.01E-06	0.032	0.011	0.02, 0.01	Island	TSS1500			
cg07328796	chr2		2.14E-06	0.033	−0.020	−0.01, −0.03	OpenSea			TRUE	
cg18696495	chr13	CLYBL	2.57E-06	0.038	−0.021	−0.01, −0.03	OpenSea	Body		TRUE	
cg04239375	chr9	NIPSNAP3B	2.67E-06	0.038	0.008	0.01, 0.00	Island	Body			
cg08273640	chr4	TBC1D14	2.91E-06	0.039	0.024	0.03, 0.01	N_Shore	TSS1500			TRUE
cg19657351	chr6	GCNT2	3.01E-06	0.039	0.015	0.02, 0.01	OpenSea	TSS200		TRUE	
cg00412337	chr6	CLIC5	3.07E-06	0.039	0.019	0.03, 0.01	N_Shore	Body		TRUE	
cg11124080	chr10	EMX2OS	3.63E-06	0.044	0.010	0.01, 0.01	Island	Body			TRUE
cg01816936	chr12	PITPNM2	4.05E-06	0.045	0.024	0.03, 0.01	OpenSea	Body			
cg07893584	chr9	PTPRD	4.13E-06	0.045	0.013	0.02, 0.01	Island	5′UTR; 1stExon; TSS200	DMR		
cg11042505	chr2		4.35E-06	0.045	−0.018	−0.01, −0.03	OpenSea			TRUE	
cg13143349	chr5		4.44E-06	0.045	0.009	0.01, 0.01	Island		DMR		
cg08463297	chr17		4.50E-06	0.045	0.025	0.04, 0.01	OpenSea			TRUE	
cg25290938	chr10	ARHGAP22	4.51E-06	0.045	0.014	0.02, 0.01	Island	TSS200		TRUE	TRUE

Among the patients, having a history of clinical depression prior to PD onset was also associated with differentially methylation regions (DMR) identified via Bumphunter in the *ChAMP* R package. Supplemental Table 2 details the DMR results. The most significant DMR was Chr1:205818668-205819609 (p = 2.0e-5, FWER = 0.007), a 941 base pair region with 9 CpGs. This region is part of the *PM20D1* gene. *PM20D1* has been associated with both PD and Alzheimer's via meta-GWAS (top PD SNP in gene has GWAS meta p-value = 6.45e-08(28,29)) and is near *NUCKS1*, another PD gene identified in the meta-GWAS (top SNP in gene meta p-value = 1.96e-16(28,29)), which we identified as important in the quantitative trait loci (QTL) analysis described below. Chr6:32145470-32146232 DMR was also suggestively associated with having a history of clinical depression prior to PD onset after multiple-testing correction (p = 2.7e-4, FWER = 0.09). This is a 762 base pair region with 28 CpGs in the *AGPAT1* gene of the major histone compatibility (MHC) class III region, which per NCBI is involved in phospholipid metabolism and metabolism gene pathways. Differential expression of the *AGPAT1* protein has previously been linked to major depression (Scifo et al., 2018).

As the PD patient population used here is unique in that they all live in a highly agricultural region on California with high levels of pesticide use, we also conducted a sensitivity analysis controlling for pesticide exposure which we have previously linked to methylation levels (Paul et al., 2018a; Furlong et al., 2020). The results were very similar with a correlation coefficient between model predicted betas at R = 0.9 (p < 2.2e-16; Supplemental Fig. 5). CpG associations for different sensitivity models can be found in Supplemental Table 1.

### 2.3 Gene set enrichment analysis

Using gene set enrichment analysis designed for methylation arrays (*methylGSEA* R package), we next assessed enrichment of KEGG, Reactome, and Panther pathways based on CpG associations with a history of clinical depression among the PD patients. Several pathways were enriched and are shown Table 3. These included multiple pathways related to immune function, KEGG pathways: Cytokine-cytokine receptor interaction (enrichment p = 8.5e-5), Osteoclast differentiation (enrichment p = 7.0e-4), Leukocyte transendothelial migration (p = 0.003); Reactome pathways: Immunoregulatory interactions between a Lymphoid and a non-Lymphoid cell (p = 3.8e-4), Neutrophil degranulation (p = 1.7e-3), Interleukin-4 and Interleukin-13 signaling (p = 0.003); Panther pathways: FAS signaling pathway (p = 0.003), Integrin signaling pathway (p = 0.006). As well as pathways related to neural function, Reactome pathways: G alpha (q) signaling events (enrichment p = 0.003); Panther pathways: Axon guidance mediated by Slit/Robo (p = 0.002), and Alzheimer disease-presenilin pathway (p = 0.02).

**Table 3:** GSEA of CpG associations from history of diagnosed depression (yes/no) EWAS among PD Patients.

	ID	Description	Size	p-value	padj
**KEGG PATHWAYS**	4060	Cytokine-cytokine receptor interaction	265	0.0001	0.004
	4380	Osteoclast differentiation	128	0.0007	0.015
	5146	Amoebiasis	106	0.0020	0.029
	4670	Leukocyte transendothelial migration	116	0.0033	0.036
	4514	Cell adhesion molecules	133	0.0057	0.050
	4510	Focal adhesion	200	0.0128	0.094
**REACTOME PATHWAYS**	R-HSA-1500931	Cell-Cell communication	116	2.38E-05	0.003
	R-HSA-198933	Immunoregulatory interactions between a Lymphoid and a non-Lymphoid cell	113	3.83E-05	0.003
	R-HSA-1474244	Extracellular matrix organization	277	8.07E-04	0.047
	R-HSA-6798695	Neutrophil degranulation	433	1.68E-03	0.074
	R-HSA-416476	G alpha (q) signalling events	200	0.0025	0.089
	R-HSA-6785807	Interleukin-4 and Interleukin-13 signaling	101	0.0030	0.089
	R-HSA-202733	Cell surface interactions at the vascular wall	127	0.0041	0.093
	R-HSA-194138	Signaling by VEGF	103	0.0045	0.093
	R-HSA-449147	Signaling by Interleukins	422	0.0048	0.093
**PANTHER PATHWAYS**	P00008	Axon guidance mediated by Slit/Robo	25	0.0022	0.115
	P00041	Metabotropic glutamate receptor group I pathway	25	0.0061	0.115
	P00020	FAS signaling pathway	34	0.0031	0.115
	P00034	Integrin signalling pathway	190	0.0059	0.115
	P00004	Alzheimer disease-presenilin pathway	124	0.0193	0.289

### 2.4 Blood-brain CpG level correlation

**Fig. 2:**
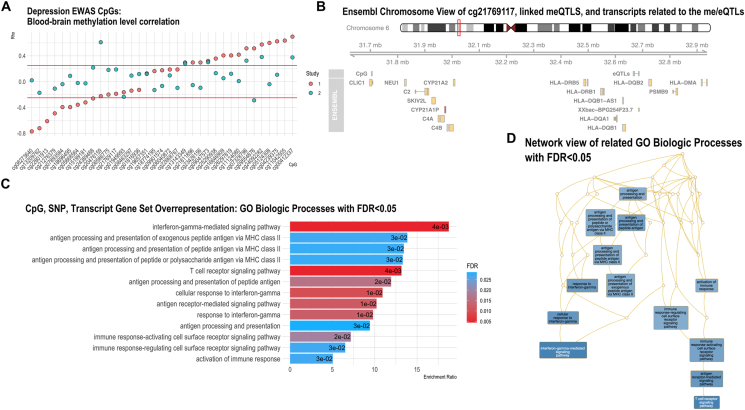
**(A)** Correlation between blood and brain methylation levels for depression EWAS CpGs. Mean correlation (absolute value to allow both positive and negative correlations to be meaningful) = 0.16, SD = 0.13. **(B)** Mapping cg21769117 (annotated as “CpG”) to its three associated me/eQTLs and the transcripts associated with the eQTLs based on GTEx expression in whole blood. **(C)** Gene Ontology (GO Biologic Processes) Gene Set Overrepresentation using all implicated genes from EWAS and QTL mapping (CpGs, SNPs, and transcripts). All biologic processes with an FDR<0.05 are shown. **(D)** Network view of overrepresented gene ontology terms (GO Biologic Processes) based on all implicated genes (CpGs, SNPs, and blood-based transcripts) versus the genome gene set.

Given the neurologic pathogenesis of PD and depression as well as the enrichment of neural function pathways, we next assessed the correlation of methylation levels in blood and brain for the depression EWAS CpGs using the Iowa Methylation Array Graphing Experiment for Comparison of Peripheral Tissue and Grey matter (IMAGE-CpG) data (n = 37; two studies) (Braun et al., 2019). Limiting this analysis to the 35 CpGs at FDR≤0.05, multiple CpGs showed both relatively high positive and negative correlations for methylation in blood and brain (Fig. 2A). Due to the small sample size of the two studies included in IMAGE-CpG (n = 12 and n = 25), the correlation estimates across CpGs varied considerably between the two studies. Nevertheless, cg00412337 (*CLIC5*), cg24092282 (intergenic), cg07328796 (intergenic), cg09047573 (*NME5*), and cg13143349 (intergenic) showed consistent correlations at R > 0.30.

### 2.5 Methylation and expression quantitative trait loci

Finally, we assessed how the EWAS-associated CpGs were related to other omic layers. First, using BIOS and our study data, we determined methylation quantitative trait loci (meQTLs, e.g. SNPs which are significantly associated with CpG levels). Second, using GTEx, we determined whether the meQTLs were also related to transcript abundance measured in both whole blood and brain. BIOS is a public database of meQTLs based on whole blood methylation, determined in 3,841 samples from five Dutch biobanks (generally age>45 years, including one longevity study with n = ∼2600 participants >89 years of age) (Bonder et al., 2017). GTEx provides characterization of genetic associations with gene expression based on 838 individuals [mean age 53.4 (range 21–70)], 52 tissues, and two cell lines (Aguet et al., 2017; Lonsdale et al., 2013).

We determined that amongst the 35 CpGs at FDR≤0.05, 25 were associated with 45 different meQTLs (Supplemental Table 3 and Table 4). This indicates that 45 SNPs (i.e. meQTLs) predicted the levels of 25 of the depression EWAS CpGs. Using GTEx, we determined 26 of these meQTLs were also expression QTLs (eQTLs), as the SNP was also associated with blood-based transcript abundance of 53 different gene transcripts (Table 4). For instance, methylation levels of cg26297819 in the transcription start site (TSS200) of the *TEF* gene was significantly associated with rs202637, a nearby intergenic SNP, based on both BIOS and PEG data (SNP-CpG p-value of 7.9e-7). rs202637 was also significantly related to the transcript abundance of TEF measured in blood (SNP-transcript p-value = 7.98e-28), as well as three other transcripts (MEI1, DESI1, and PMM1; Table 4). Interestingly, rs202637 is associated with PD (meta-GWAS p < 0.05 (Lill et al., 2012; Nalls et al., 2014)) and *TEF* polymorphisms have been linked with depression and depression in PD specifically (Kripke et al., 2009; Hua et al., 2012).

**Table 4:** History of clinical depression EWAS CpGs and Functional Genetics. QTL analysis, methylation QTLs (meQTL) SNPs → CpGs (Dutch Biobank) and SNPs →expression (GTEx). Expression based on transcripts in whole blood from GTEx.

CpG	EWAS p-value	CpG Gene	CpG Chr	CpG Group	Type	SNP	SNP-CpG P-valueƚ	SNP Chr	SNP Chr. Position	SNP Gene	Type	SNP-transcript P-value (GTEx)	SNP to TSS Distance	Transcript Gene	HGNC Name^a^
cg18774195	2.07E-08	SLC7A14	3	5′UTR; 1stExon	NA										
cg26297819	1.14E-07	TEF	22	TSS200	cis-meQTL	rs202637 ƚ	7.93E-07	22	41853928		eQTL	2.11E-46	−241575	MEI1	meiotic double-stranded break formation protein 1
											eQTL	7.98E-28	90591	TEF	TEF transcription factor, PAR bZIP family member
											eQTL	3.21E-19	−163172	DESI1	desumoylating isopeptidase 1
											eQTL	4.66E-19	−131966	PMM1	phosphomannomutase 1
cg09047573	1.18E-07	NME5	5	5′UTR	cis-meQTL	rs7734861	3.31E-13	5	137507979	BRD8	eQTL	1.89E-80	−438677	CTNNA1	catenin alpha 1
cg16646909	1.36E-07	ZNF790	19	TSS1500	cis-meQTL	rs2445878	5.23E-34	19	37318357	ZNF790	eQTL	6.03E-04	159353		ENSG00000276071
	1.36E-07				cis-meQTL	rs1227799	1.66E-16	19	37289551	ZNF790-AS1	NA				
cg01949993	1.71E-07	LAMC2	1	TSS200	cis-meQTL	rs6678888	9.05E-70	1	183155700	LAMC2	eQTL	8.51E-34	163105	LAMC1	laminin subunit gamma 1
					cis-meQTL	rs76831965	1.92E-08	1	183119724		eQTL	6.72E-03	−321626	SMG7-AS1	SMG7 antisense RNA 1
					cis-meQTL	rs875792	1.29E-05	1	183185784	LAMC2	NA				
cg21769117	2.12E-07	CLIC1	6	TSS1500	trans-meQTL	rs548987	3.25E-08	6	25869371	SLC17A3	eQTL	8.42E-119	−496016	BTN3A2	butyrophilin subfamily 3 member A2
											eQTL	2.01E-11	−93659	TRIM38	tripartite motif containing 38
											eQTL	1.86E-09	−123519		ENSG00000272462
					cis-meQTL	rs1794282	6.21E-06	6	32666526		eQTL	5.74E-161	30366	HLA-DQB1	major histocompatibility complex, class II, DQ beta 1
											eQTL	2.35E-111	168462	HLA-DRB5	major histocompatibility complex, class II, DR beta 5
											eQTL	7.37E-66	−155412	PSMB9	proteasome subunit beta 9
											eQTL	3.50E-56	70570	HLA-DQA1	major histocompatibility complex, class II, DQ alpha 1
											eQTL	3.74E-51	38394	HLA-DQB1-AS1	HLA-DQB1 antisense RNA 1
											eQTL	1.17E-48	739638	SKIV2L	Ski2 like RNA helicase
											eQTL	1.29E-30	716725	C4A	complement C4A (Rodgers blood group)
											eQTL	2.57E-29	683987	C4B	complement C4B (Chido blood group)
											eQTL	1.15E-28	108901	HLA-DRB1	major histocompatibility complex, class II, DR beta 1
											eQTL	3.87E-18	693060	CYP21A1P	cytochrome P450 family 21 subfamily A member 1, pseudogene
											eQTL	1.30E-15	660484	CYP21A2	cytochrome P450 family 21 subfamily A member 2
											eQTL	2.34E-04	−270345	HLA-DMA	major histocompatibility complex, class II, DM alpha
											eQTL	7.34E-04	800964	C2	complement C2
											eQTL	7.63E-03	958986	CLIC1	chloride intracellular channel 1
											eQTL	3.34E-02	835843	NEU1	neuraminidase 1
					cis-meQTL	rs1613056	1.05E-05	6	32668946		eQTL	5.74E-161	32786	HLA-DQB1	major histocompatibility complex, class II, DQ beta 1
											eQTL	3.76E-147	−62365	HLA-DQB2	major histocompatibility complex, class II, DQ beta 2
											eQTL	2.35E-111	170882	HLA-DRB5	major histocompatibility complex, class II, DR beta 5
											eQTL	7.37E-66	−152992	PSMB9	proteasome subunit beta 9
											eQTL	3.50E-56	72990	HLA-DQA1	major histocompatibility complex, class II, DQ alpha 1
											eQTL	3.74E-51	40814	HLA-DQB1-AS1	HLA-DQB1 antisense RNA 1
											eQTL	1.17E-48	742058	SKIV2L	Ski2 like RNA helicase
											eQTL	1.29E-30	719145	C4A	complement C4A (Rodgers blood group)
											eQTL	2.57E-29	686407	C4B	complement C4B (Chido blood group)
											eQTL	1.15E-28	111321	HLA-DRB1	major histocompatibility complex, class II, DR beta 1
											eQTL	3.87E-18	695480	CYP21A1P	cytochrome P450 family 21 subfamily A member 1, pseudogene
											eQTL	1.30E-15	662904	CYP21A2	cytochrome P450 family 21 subfamily A member 2
											eQTL	2.34E-04	−267925	HLA-DMA	major histocompatibility complex, class II, DM alpha
cg20476159	2.71E-07	CTDP1	18	Body	cis-meQTL	rs111832832	3.64E-15	18	77454511	CTDP1	eQTL	5.20E-102	14710	CTDP1	CTD phosphatase subunit 1
					cis-meQTL	rs11660714	9.33E-05	18	77494911	CTDP1	eQTL	5.20E-102	55110	CTDP1	CTD phosphatase subunit 1
					cis-meQTL	rs7238206	3.27e-310	18	77444285	CTDP1	eQTL	5.20E-102	4484	CTDP1	CTD phosphatase subunit 1
					cis-meQTL						eQTL	4.43E-29	4927		ENSG00000274828
cg13209762	4.17E-07		1		cis-meQTL	rs2000239	2.31E-53	1	53883126	SLC25A3P1	NA				
cg24092282	4.27E-07		14		cis-meQTL	rs1137724	1.00E-08	14	54408633		NA				
cg06040872	4.75E-07	CCL18	17	Body	NA										
cg09901574	5.18E-07		16		NA										
cg09554876	5.61E-07	BEND5; AGBL4	1	Body; Body	NA										
cg23426156	6.13E-07		8		NA										
cg21811896	6.81E-07	MEGF6	1	Body	NA										
cg22668767	7.32E-07		5		cis-meQTL	rs334881	4.71E-66	5	54520702	MCIDAS	NA				
					cis-meQTL	rs76925544	4.83E-05	5	54767838	PLPP1	NA				
cg15199181	7.92E-07		1		cis-meQTL	rs823114 ƚƚ	3.27e-310	1	205719532		eQTL	1.50E-79	−99728	PM20D1	peptidase M20 domain containing 1
											eQTL	2.38E-35	−25056	RAB29	RAB29, member RAS oncogene family
											eQTL	3.79E-13	−63344	SLC41A1	solute carrier family 41 member 1
											eQTL	3.48E-07	128	NUCKS1	nuclear casein kinase and cyclin dependent kinase substrate 1
					cis-meQTL	rs73080333	1.96E-44	1	205717676	NUCKS1	eQTL	1.50E-79	−101584	PM20D1	peptidase M20 domain containing 1
											eQTL	2.38E-35	−26912	RAB29	RAB29, member RAS oncogene family
					cis-meQTL	rs1772145 ƚ	1.22E-08	1	205695695	NUCKS1	eQTL	1.50E-79	−123565	PM20D1	peptidase M20 domain containing 1
					cis-meQTL	rs16856462	8.27E-07	1	205887981	SLC26A9	NA				
cg06586775	8.01E-07	SPAG4	20	Body	cis-meQTL	rs2425151	3.24E-38	20	34394541	PHF20	eQTL	1.36E-190	141860	CPNE1	copine 1
											eQTL	1.20E-52	261872		ENSG00000224497
											eQTL	1.31E-16	394478	UQCC1	ubiquinol-cytochrome c reductase complex assembly factor 1
cg05124308	1.24E-06	TMEM200B	1	TSS200	cis-meQTL	rs12097553 ƚ	4.70E-05	1	29571222	PTPRU	eQTL	3.46E-05	357619	EPB41	erythrocyte membrane protein band 4.1
cg05868564	1.38E-06		11		NA										
cg22801913	1.65E-06	C11orf49	11	Body	cis-meQTL	rs3740694	1.22E-11	11	47182926	C11orf49	eQTL	1.62E-22	−191327	MYBPC3	myosin binding protein C, cardiac
					cis-meQTL	rs326224	3.26E-06	11	47255598	DDB2	eQTL	5.88E-26	−14859	ACP2	acid phosphatase 2, lysosomal
cg24389488	1.74E-06	WNT3	17	Body	NA										
cg21276379	2.01E-06	EPAS1	2	TSS1500	cis-meQTL	rs13428739	9.18E-99	2	46523934	EPAS1	NA				
					cis-meQTL	rs12617123	4.76E-07	2	46504962		NA				
cg07328796	2.14E-06		2		cis-meQTL	rs11888416	1.41E-05	2	227335605		NA				
cg18696495	2.57E-06	CLYBL	13	Body	cis-meQTL	rs2761154	6.58E-43	13	100443424	CLYBL	NA				
					cis-meQTL	rs8000435	1.25E-16	13	100310215	CLYBL	eQTL	1.87E-08	51292	CLYBL	citrate lyase beta like
					cis-meQTL	rs12323058	2.61E-05	13	100360104	CLYBL	NA				
cg04239375	2.67E-06	NIPSNAP3B	9	Body	cis-meQTL	rs62565987	5.62E-104	9	107525011		eQTL	9.20E-19	15042	NIPSNAP3A	nipsnap homolog 3A
					cis-meQTL	rs4149341	2.21E-22	9	107544285	ABCA1	eQTL	9.20E-19	34316	NIPSNAP3A	nipsnap homolog 3A
					cis-meQTL	rs7027419	2.72E-05	9	107485577	LOC107987105	eQTL	9.20E-19	−24392	NIPSNAP3A	nipsnap homolog 3A
cg08273640	2.91E-06	TBC1D14	4	TSS1500	cis-meQTL	rs7682697	8.47E-27	4	6906099		NA				
					cis-meQTL	rs4689053	1.10E-06	4	6909777		NA				
					cis-meQTL	rs10020189	3.54E-06	4	6907052		NA				
cg19657351	3.01E-06	GCNT2	6	TSS200	cis-meQTL	rs9393357	9.26E-18	6	10521054	GCNT2	eQTL	1.02E-29	28598	GCNT2	glucosaminyl (N-acetyl) transferase 2 (I blood group)
cg00412337	3.07E-06	CLIC5	6	Body	NA										
cg11124080	3.63E-06	EMX2OS	10	Body	trans-meQTL	rs9266629	2.30E-07	6	31346822		eQTL	6.54E-119	−16450		ENSG00000272221
											eQTL	1.74E-55	106940	HLA-C	major histocompatibility complex, class I, C
											eQTL	7.73E-46	201146		ENSG00000204528
											eQTL	7.73E-28	181008		ENSG00000272501
											eQTL	3.70E-11	−213940	NCR3	natural cytotoxicity triggering receptor 3
											eQTL	1.73E-09	−811141	PBX2	PBX homeobox 2
cg01816936	4.05E-06	PITPNM2	12	Body	cis-meQTL	rs641760	1.46E-07	12	123518866	PITPNM2	eQTL	1.33E-42	−238015	CDK2AP1	cyclin dependent kinase 2 associated protein 1
											eQTL	5.63E-11	−349454	KMT5A	lysine methyltransferase 5A
											eQTL	1.04E-07	52669	ARL6IP4	ADP ribosylation factor like GTPase 6 interacting protein 4
											eQTL	1.59E-04	52749	ABCB9	ATP binding cassette subfamily B member 9
cg07893584	4.13E-06	PTPRD	9	5′UTR; 1stExon; TSS200	cis-meQTL	rs5013501	2.83E-05	9	10665525		NA				
cg11042505	4.35E-06		2		NA										
cg13143349	4.44E-06		5		cis-meQTL	rs11959614	3.25E-08	5	1930990		NA				
cg13143349	4.44E-06		5		cis-meQTL	rs56287698	2.84E-05	5	1958907	ENSG00000248994	NA				
cg08463297	4.50E-06		17		NA										
cg25290938	4.51E-06	ARHGAP22	10	TSS200	cis-meQTL	rs17010948	5.48E-79	10	49812917	ARHGAP22	NA				
					cis-meQTL	rs10857612 ƚ	2.90E-12	10	49824141	ARHGAP22	eQTL	5.86E-28	−40169	ARHGAP22	Rho GTPase activating protein 22

NA: Not a applicable (CpG not associated to meQTL, or meQTL is not associated with transcript adundance and not also an eQTL.

PDGene (meta-GWAS): ƚ meta p < 0.05; ƚƚ meta p = 1.78E-13.

a Ensembl_id when HGNC unavailable.

cg15199181, an intergenic CpG, was associated with four meQTLs, most strongly rs823114 (SNP-CpG p-value = 3.27E-310). Not only was rs823114 also picked up in the PD meta-GWAS (p = 1.78E-13 (Lill et al., 2012; Nalls et al., 2014)), but this SNP was also related to blood-based transcript levels of four different genes, *PM20D1*, *RAB29*, *SLC41A1*, and *NUCKS1* based on GTEx. One final notable CpG was cg21769117 in the *CLIC1* gene transcription start site (TSS1500), which is located in the MHC class III region. This CpG was significantly associated with three meQTLs, SNPs which were also eQTLs associated with the abundance of 18 different transcripts from genes in the region, many immune system related, including HLA genes and complement system protein genes. cg21769117 and its associated SNP and transcript network are mapped onto the chromosome in Fig. 2B. Supplemental Fig. 6 shows all genes in the dense chromosomal region where cg21769117 and the me/eQTLs are located. This figure demonstrates that the me/eQTLs were not associated with transcript abundance of every gene in the region surrounding cg21769117, but instead selectively associated with the 18 specific transcripts.

To synthesize the network of associated CpG, SNP, and transcripts, we further took the entire set of implicated genes (79 genes with an associated CpG from the EWAS, SNP via meQTL, or transcript associated with the me/eQTLs in blood) and performed gene set overrepresentation analysis to test for overrepresentation of biologic processes. The overrepresented (FDR<0.05) gene ontology (GO) biologic processes are shown in Supplemental Table 4 and Fig. 2C. Given the large number of transcripts from the MHC class III region associated with the three me/eQTLs linked to cg21769117, immune system processes were strongly overrepresented, most significantly: interferon-gamma-mediated signaling pathway (enrichment ratio = 18.8, FDR = 4.4e-03); T cell receptor signaling pathway (enrichment ratio = 13.2, FDR = 4.4e-03); antigen receptor-mediated signaling pathway (enrichment ratio = 10.9, FDR = 0.01). Many of the associated biologic processes are related processes, shown in a graph of enriched GO terms that displays the connections between the GO terms (Fig. 2D).

Interestingly, of 45 me/eQTLs that were associated with abundance of 53 different transcripts in blood, 14 were also associated with the abundance of 22 different transcripts in one of three brain regions of interest: the substantia nigra, putamen basal ganglia, and frontal cortex (Table 5). Notably, fewer transcripts in the MHC class III region were related to the eQTLs (8 in the brain versus 18 in blood). There was also differential abundance of three transcripts, CYP21A1P, RAB29, and CPNE1, in the substantia nigra specifically related to three eQTLs. The PD GWAS SNP rs823114, which was associated with cg15199181, was also linked to expression of RAB29 in all three regions (substantia nigra, putamen basal ganglia, and frontal cortex) and PM20D1 in the frontal cortex.

**Table 5:** History of clinical depression EWAS CpGs and Functional Genetics. QTL analysis, methylation QTLs (cis-meQTL) SNPs → CpGs (Dutch Biobank) and SNPs →expression (GTEx). Expression based on transcripts in samples from three brain regions (putamen basal ganglia, frontal cortex, and substantia nigra) from GTEx.

CpG	EWAS p-value	CpG Gene	CpG Chr	meQTL Type	SNP	SNP-CpG meQTL p-value	Dir. of SNP-CpG beta	SNP Gene	SNP Chr	SNP to TSS Distance	Dir. of SNP-expression beta	Putamen Basal Ganglia eQTL P-value	Frontal Cortex eQTL P-value	Substantia Nigra eQTL P-value	Symbol	Name
cg26297819	1.14E-07	TEF	22	cis	rs202637 ƚ	7.93E-07	+		22	−102839	–	3.84E-09	2.96E-18		CSDC2	cold shock domain containing C2
										−86682	–	2.04E-08	5.87E-11		POLR3H	RNA polymerase III subunit H
										−241575	–		3.80E-06		MEI1	meiotic double-stranded break formation protein 1
cg16646909	1.36E-07	ZNF790	19	cis	rs2445878	5.23E-34	–	ZNF790	19	254385	+	1.15E-23	3.65E-16		ZNF529-AS1	ZNF529 antisense RNA 1
				cis	rs1227799	1.66E-16	+	ZNF790-AS1	19	−453263	+	1.80E-40	2.77E-44		LINC01535	long intergenic non-protein coding RNA 1535
										−708332	+	5.26E-03			ZNF793-AS1	ZNF529 antisense RNA 1
cg01949993	1.71E-07	LAMC2	1	cis	rs6678888	9.05E-70	–	LAMC2	1	327	+	3.94E-09	4.71E-10		LAMC2	laminin subunit gamma 2
cg21769117	2.12E-07	CLIC1	6	trans	rs548987	3.25E-08	–	SLC17A3	6	−496016	–		3.17E-19		BTN3A2	butyrophilin subfamily 3 member A2
										−123519	+	9.07E-09				ENSG00000272462.2
				cis	rs1794282	6.21E-06	–		6	30366	–		1.50E-20		HLA-DQB1	major histocompatibility complex, class II, DQ beta 1
										716725	–	8.58E-06	1.16E-09		C4A	complement C4A (Rodgers blood group)
										693060	–	3.76E-07	5.39E-08		CYP21A1P	cytochrome P450 family 21 subfamily A member 1, pseudogene
										520395	–		4.67E-05		RNF5	ring finger protein 5
										958801	–	4.98E-03	6.19E-03		MSH5	mutS homolog 5
				cis	rs1613056	1.05E-05	–		6	32786	–	3.52E-28	1.50E-20		HLA-DQB1	major histocompatibility complex, class II, DQ beta 1
										719145	–	8.58E-06	1.16E-09		C4A	complement C4A (Rodgers blood group)
										695480	–	3.76E-07	5.39E-08	4.20E-05	CYP21A1P	cytochrome P450 family 21 subfamily A member 1, pseudogene
										662904	+	5.30E-05	1.49E-02		CYP21A2	cytochrome P450 family 21 subfamily A member 2
cg22668767	7.32E-07		5	cis	rs334881	4.71E-66	–	MCIDAS	5	−8806	–	8.13E-20	3.59E-12		CCNO	cyclin O
								PLPP1		−2441	–	2.99E-03			MCIDAS	multiciliate differentiation and DNA synthesis associated cell cycle protein
cg15199181	7.92E-07		1	cis	rs823114 ƚƚ	3.27e-310	–		1	−99728	–		2.54E-09		PM20D1	peptidase M20 domain containing 1
										−25056	+	1.95E-05	2.02E-03	1.28E-03	RAB29	RAB29, member RAS oncogene family
cg06586775	8.01E-07	SPAG4	20	cis	rs2425151	3.24E-38	+	PHF20	20	141860	–	9.66E-25	1.22E-25	1.23E-11	CPNE1	copine 1
				cis	rs326224	3.26E-06	+	DDB2	11	−352646	+		1.41E-05		FAM180B	family with sequence similarity 180 member B
				cis	rs4149341	2.21E-22	–	ABCA1	9	34316	–	6.90E-10	5.23E-04		NIPSNAP3A	nipsnap homolog 3A
				cis	rs7027419	2.72E-05		LOC107987105	9	34316	+	6.90E-10			NIPSNAP3A	nipsnap homolog 3A
cg01816936	4.05E-06	PITPNM2	12	cis	rs641760	1.46E-07	+	PITPNM2	12	−226651	+	3.06E-04				ENSG00000235423.8

PDGene (meta-GWAS): ƚ meta p < 0.05; ƚƚ meta p = 1.78E-13.

## 3 Discussion

Depression is highly prevalent in Parkinson's disease (PD) (Kasten et al., 2010). Yet, the etiology of this important non-motor feature, while likely multifactorial, is not well understood (Simuni and Sethi, 2008). To assess biologic disruptions associated with depression in PD, we used a population-based study of patients early in disease course and employed blood-based methylation to relate leukocyte composition and genome-wide white blood cell methylation levels to two indicators of depression: having a history of clinical depression and current depressive symptoms. Although the two depression measures were moderately correlated (R = 0.32), indicating some patients had both a history of clinical depression and current depressive symptoms, each indicator was associated with different methylation patterns. Nevertheless, both implicated immune system function involvement.

We first characterized PD patient immune cell profiles via immunomethylomics and methylation profiling (Kelsey and Wiencke, 2018). Levels of several leukocyte indicators suggested more immune activation in patients with current depressive symptoms than in either PD patients or controls without current depressive symptoms. This was perhaps most comprehensively shown by the neutrophil-to-lymphocyte ratio (NLR). The NLR, which is simply a ratio of lymphocyte to neutrophil counts, here measured with epigenetic surrogates as leukocyte markers, is often assessed as a measure of systemic inflammation and immune activation (Song et al., 2021). It reflects the balance between inflammation (acute or chronic), as measured by neutrophil levels, and adaptive immunity, measured by lymphocyte levels (Song et al., 2021). Interestingly, even our controls were showing an average NLR of 2.1, which is just below the “grey zone” of 2.3–3.0 that indicates immune activation and perhaps early signs of pathologic states (Zahorec, 2021). This likely reflects our aged study population, as age strongly predicts decreased immune function, increased immune activation, and a higher NLR(44,45). Yet, the PD patients experiencing current depressive symptoms showed a markedly higher NLR on average (3.4) than either controls (2.1) or PD patients without depressive symptoms (2.8). Furthermore, the patients with current depressive symptoms also showed more accelerated epigenetic immune system aging, which is an indicator of biologic aging and inflamm-aging. We have previously linked other immune system aging markers to PD as well (Paul et al., 2021). However, the current study demonstrates even more pronounced increases for PD patients experiencing current depressive symptoms. Interestingly, PD patients with a history of clinical depression prior to PD diagnosis had immune cell profiles similar to PD patients without a history of clinical depression. This result seems to indicate that the differences in leukocyte composition of the blood are related to current depressive symptoms and health states rather than reflecting a lasting pathology of clinical depression without current symptoms.

Although current depressive symptoms were necessary to see activated immune cell composition profiles, PD patients with a history of clinical depression showed differences in CpG levels across the genome that were unrelated to blood cell composition. We identified 35 specific CpGs associated at an FDR<0.05 and 569 at a relaxed significance threshold for discovery (FDR<0.10). Several of the CpGs are noteworthy, including cg23426156, cg11042505, and cg21769117. Two, cg23426156 and cg11042505, have been linked to the important neuropsychiatric disorder schizophrenia in three different study populations (Hannon et al., 2016, 2021). While cg23426156 is intergenic, it is in the transcription factor binding site for several factors (i.e., CEBPB, STAT3, JunD, GR, RFX5_(N-494), Rad21, p300_(N-15), SMC3_(ab9263), Pol2-4H8, c-Fos). The CpG cg21769117 on the other hand is in the transcription start site region of the gene *CLIC1*. *CLIC1* and the chloride intracellular channel 1 (CLIC1) protein have been compellingly linked to neurodegeneration. For instance, during chronic inflammatory states in the central nervous system, CLIC1 increasingly accumulates in peripheral blood mononuclear cells, as shown in Alzheimer's patients (Carlini et al., 2020). Proteomics analysis of plasma from PD patients and controls also found higher levels of CLIC1 in PD patients (Dong et al., 2019). CLIC1 has also been implicated in microglia-mediated β-amyloid peptide neurotoxicity (Skaper et al., 2013) and IL-1β biology (Domingo-Fernández et al., 2017), connecting the gene and its protein with inflammatory neurodegenerative processes. Our pathway and enrichment analyses further implicated several immune function pathways with a history of clinical depression among the patients, including among KEGG pathways, cytokine-cytokine receptor interaction and leukocyte transendothelial migration, according to Reactome pathways, immunoregulatory interactions between a lymphoid and a non-lymphoid cell, interleukin-4 and interleukin-13 signaling, and neutrophil degranulation, and from Panther pathways, FAS (death receptor signaling on cytotoxic T cells and NK cells) and Integrin (principal cell adhesion receptors that mediate leukocyte migration and activation) signaling pathways.

To assess how these CpGs identified as associated with a history of depression in PD may be related to other biologic layers, we further linked CpGs to quantitative trait loci and determined whether meQTLs were eQTLs based on GTEx. We found that 45 single nucleotide polymorphisms (SNPs) were associated with 25 of the 35 CpGs associated at FDR<0.05. Furthermore, many of these methylation QTLs were also eQTLs such that the SNP variant was related to both methylation levels of one of the EWAS CpGs and expression-based abundance of different transcripts measured in both blood and brain regions. This is expected, as many if not most cis-eQTLs occur at the same genomic location as a cis-meQTL(50). One of the best described functions of methylation is regulating gene expression (Moore et al., 2013) and our QTL analysis seems to support this, closely linking genetic variation, methylation, and expression. For instance, one of the top hits, cg26297819, is in the transcription start site region of *TEF*. One meQTL was associated with the CpG, rs202637. Not only has this SNP previously been associated with PD in a meta-GWAS (p < 0.05 (Lill et al., 2012; Nalls et al., 2014)), but *TEF* variants have been associated with depression in PD as well (Kripke et al., 2009; Hua et al., 2012). The meQTL was also strongly related to transcript abundance of not only TEF measured in blood, but also CSDC2, POLR3H, and MEI1 in brain, transcripts which have been linked to depression in a large meta-GWAS(51).

The differentially methylated region (DMR) and QTL analysis also converged on two regions of relevance for our outcome. First, having a clinical history of depression among the PD patients was related to a DMR in chromosome 1, in a genetic region near *PM20D1*, *SLC41A1*, *RAB29*, and *NUCKS1*. The most significant individual CpG related to depression in the region we identified was cg15199181 (FDR = 0.02). This CpG was associated with three meQTLs, most significantly rs823114. This SNP, in the *NUCKS1* gene, was strongly associated with PD in the meta-GWAS (meta p-value = 1.78E-13 (Lill et al., 2012; Nalls et al., 2014)). Furthermore, this meQTL is also an eQTL associated with expression levels of *PM20D1*, *RAB29*, *SLC41A1*, and *NUCKS1* in blood, *RAB29* in the basal ganglia, frontal cortex, and substantia nigra, and PM20D1 in the frontal cortex, i.e., brain areas of interest related to dopamine signaling. Several of these factors have been linked to PD experimentally. For instance, Rab29 has been shown to activate the Parkinson's-associated LRRK2 kinase (Bonet-Ponce and Cookson, 2019; Purlyte et al., 2018). This association supports the notion that PD genetic risk loci may also influence symptom profiles among patients, which we have previously reported (Paul et al., 2018b) and also observe here with the PD GWAS SNP rs823114 influencing the methylation levels of cg15199181. Importantly, our findings suggest that part of the genetic risk may be conferred through an influence on mechanisms involving methylation and expression levels.

A history of clinical depression in PD was also related to a second DMR in chromosome 6 in the MHC class III region, near cg21769117, the CpG in the *CLIC1* gene. The CpG was associated with four meQTLs that are also eQTLs associated with expression levels of 18 different transcripts in blood and 9 different transcripts in the brain (basal ganglia, frontal cortex, or substantia nigra). These transcripts include CLIC1, many other immune related proteins (HLAs, complement proteins), and notably *CYP21A1P* expression in the substantia nigra, which we determined based on our eQTL analysis with GTEx. cg00412337 in the nearby *CLIC5* gene was also positively associated with depression. While these results are interesting, the complexity of the MHC class III region with hundreds of genes, requires additional evidence before drawing conclusions about its effects on depression in PD.

When we considered the complete gene set from all implicated CpGs, QTLs, and blood-based transcripts, all enriched biologic processes were related to immune function (e.g., interferon-gamma-mediated signaling pathway, antigen processing and presentation pathways, and immune response-activating pathways). Many of the 21 transcripts expressed in the brain regions were also related to lymphocyte mediated immunity and adaptive immune response, along with biosynthesis of mineralocorticoids and glucocorticoids via *CYP21A2* and its pseudogene *CYP21A1P*. Glucocorticoids are immunoregulatory hormones generally synthesized in the adrenal cortex, where most *CYP21A2*/*CYP21A1P* expression occurs (Ahmed et al., 2019). However, glucocorticoid synthesis has been observed in the brain (Ahmed et al., 2019), seemingly allowing local regulation of immunologic response, and the GTEx analysis indicates both *CYP21A2* and *CYP21A1P* are expressed in the basal ganglia, frontal cortex, and substantia nigra, with some variation in expression determined by eQTLs of interest. Furthermore, *CYP21A1P* is a transcribed pseudogene, or ancestral copy of the protein-coding gene that are thought to have lost the functional product of their parental gene due to accumulation of mutations (Milligan and Lipovich, 2015). While not all pseudogenes are transcribed, for those that are, they can provide a key mechanism for regulating the parental gene's expression (Milligan and Lipovich, 2015; Li et al., 2013). Overall, this differentially methylated region associated with clinical depression in PD seems to have genetic contributors (meQTLs/eQTLs) related to expression of important immune genes both peripherally and centrally.

Ultimately, our analysis demonstrates the dynamic and interdependent nature of biologic systems. Our EWAS linked distinct CpGs to depression among PD patients, functional genomics and QTL mapping broadened the scope of our investigation and strongly implicated the immune system in neurodegeneration and depression in PD. In the future, multi-omics measurements from the same study participants may allow us to link these QTLs and methylation levels to measured transcripts specifically in PD patients. This analysis also suggests that blood is an important tissue that provides clues into mechanisms that contribute to depression and neurodegenerative disease. Our analysis strongly implicated immune system function as being disrupted among PD patients with a history of clinical depression as suggested by differential leukocyte methylation.

Overall, the current study maps methylation signals associated with depression among Parkinson's disease. The findings provide evidence of immune system involvement in depression among Parkinson's patients, both for those experiencing current depressive symptoms and those with a history of clinical depression. The first expressed in leukocyte composition and the second in methylation level differences in specific immune system related chromosomal areas. These may be both a consequence of disease pathogenesis and a contributor to its progression. Future research should investigate whether such signals are specific to PD or also related to depression in other neurodegenerative disorders, such as Alzheimer's.

PD is a highly heterogenous disorder with patients experiencing different symptom profiles, often including depression. Comprehensively mapping biologic pathways and perturbations associated with distinct symptoms may shed light onto PD's complex clinical heterogeneity and etiology.

## 4 Methods

### 4.1 Study population

This study was based on participants of the Parkinson's disease, Environment, and Genes (PEG) study (Narayan et al., 2013), a population-based PD study from three counties in California's Central Valley (Kern, Fresno, and Tulare). PEG was designed as a case-control study to investigate PD etiology (2001–2007 & 2010–2016; n = 849 PD patients early in disease; n = 1021 population-based controls). Further information on the patient population has been published (Duarte Folle et al., 2019). Informed consent was obtained from all subjects and the study was approved by the UCLA institutional review board. For this analysis, data were restricted to 465 PD patients and 227 controls of European ancestry with methylation data. All patients in the study were examined by movement disorder specialists (lead by J.B.) at least once and confirmed as having probable idiopathic PD based on published criteria (Hughes et al., 1992a, 1992b). Demographic characteristics for PD patients with and without methylation data were similar, including average age 70.4 (SD = 11.7) vs 70.5 (SD = 9.8) and 62% vs 65% male. We assessed two indicators of depression, first, having a history of clinical depression. This indicator was based on self-reported physician diagnosis during interviews conducted by trained study staff. Second, we assessed current depressive symptoms at the time of blood draw, measured by a self-administered 15-item geriatric depression scale (GDS).

### 4.2 Methylation and QTL analysis

For methylation, DNA was extracted from peripheral whole blood at the baseline visit for all study participants. We profiled and processed DNA samples using the Illumina Infinium 450k platform (486k CpGs) with standard settings. We used k nearest neighbors for imputation from the Impute R package, the background normalization method from the Genome Studio software to process DNA methylation β values, and corrected for type I/type II probe bias with BMIQ using the champ. norm function in the ChAMP R package (Morris et al., 2014). For analysis we removed x-reactive CpGs (29,233 CpGs), CpGs that had a SNP in the probe (probe_rs), CpG interrogation site (CpG_rs), or the single nucleotide extension (SBE_rs) (104,206 CpGs), and CpGs that were in the X or Y chromosome (11,648 CpGs). Analysis was therefore based on 352,325 CpGs. More detail has been published (Chuang et al., 2017; Paul et al., 2021) and the data are available on Gene Expression Omnibus (GEO), accession numbers GSE72774 and GSE72776.

We estimated whole blood cell composition from the DNAm using the Houseman estimation method to estimate the proportion of CD8^+^ T cells, CD4^+^ T cells, natural killer, B cells, monocytes, neutrophils, and eosinophils (Houseman et al., 2012, 2014). The neutrophil to lymphocyte ratio was calculated by taking the ratio of neutrophils to lymphocytes (CD8^+^ T, CD4^+^ T, natural killer, and B cells). We also estimated DNAm epigenetic immune age acceleration using extrinsic epigenetic age acceleration (EEAA). EEAA is a measure of biologic aging in immune cells that is based on the Hannum clock (Hannum et al., 2013), but dependent on white blood cell concentrations (Horvath and Levine, 2015).

To establish meQTLs associated with the EWAS CpGs, we used two sources, first, the BIOS public database of meQTLs, which is based on whole blood methylation from 3,841 samples from five Dutch biobanks (Bonder et al., 2017). Second, we used meQTLs established in our PEG PD study population. SNP data in PEG were derived from genome-wide association data generated using the Global Screening Array (Illumina, Inc.) and subjected to standard quality control and genotype imputation by Minimac3(64). For details on the genotyping and post-genotyping procedures applied to the PEG dataset see ref (Hong et al., 2020). To establish the meQTLs, we limited to the 17 EWAS CpGs (Table 2) and ran pair-wise linear regression models (i.e., every CpG ∼ SNP pair) among those of European ancestry, controlling for age, sex, and AIMs fractional ancestry. CpG ∼ SNP pairs with an FDR<0.05 were considered meQTLs. Two additional meQTLs were determined from this analysis. The majority of meQTLs were detected in both study populations (Supplemental Table 3).

To establish if the meQTL was also an eQTL, we used GTEx v8, which provides characterization of genetic associations and gene expression and splicing in 838 individuals, 52 tissues, two cell lines (whole-genome sequence and RNA-sequence from approximately 960 deceased adult donors; 85% European Ancestry, 66% male, mean age 53.4 (21–70), mean BMI 27.3 (both men and women)) (Aguet et al., 2017; Lonsdale et al., 2013). As expression is tissue-dependent, we determined eQTLs based on whole-blood and three brain regions of importance in PD: substantia nigra, putamen basal ganglia, and frontal cortex.

### 4.3 Statistical analysis

Mean differences in cell composition markers between groups (PD patients with depression versus PD patients without depression and controls without depression) were compared with a Wilcoxon test. Using logistic regression models controlling for covariates listed below, we confirmed the mean comparisons, i.e., associations remained after confounder adjustment. For the epigenome-wide association analysis (differentially methylation positions, DMP), we used the *meffil* R package to test for association between the binary depression indicators and each CpG site using linear regression models fit with *limma* (Ritchie et al., 2015). To control for potential confounding, we included the following covariates: age at blood draw, sex, smoking, number of years with PD at blood draw, AIMs-based Caucasian fractional ancestry, an indicator for PEG study wave, and blood cell composition (CD4T and neutrophil proportions). We also included an indicator for pesticide exposure in sensitivity analysis. With the EWAS DMP associations, we next performed gene set enrichment analysis using the *methylGSA* R package, which accounts for the number of CpGs per gene included the Illumina 450k array. We assessed enrichment of KEGG pathways, Reactome pathways, and Panther pathways. Multiple testing for both the DMP EWAS and GSEA was *adjusted* for with a false discovery rate (FDR). To assess differentially methylated regions (DMR), we used the *ChAMP* R package, applying bumphunter and 1000 bootstraps to estimate regions for which the methylation genomic profile deviates between groups. The DMR function detects differentially methylation regions between two populations (i.e., patients with and without depression), and returns the DMRs and estimated p-values. Multiple testing was adjusted for with a family-wise error rate (FWER). Finally, for gene set enrichment based on all genes implicated by the EWAS and QTL analysis, we used WebGestalt (WEB-based Gene Set Analysis Toolkit), implemented via R. We assessed over-representation of the gene set for gene ontology terms and network topology-based analysis.

## Ethics approval and consent to participate

The PEG study was approved by the UCLA Institutional Review Board (IRB# 11–001530) and informed consent was obtained from all individuals. Our research conformed to the Declaration of Helsinki.

## Availability of data and materials

The DNA methylation data is available in the GEO repository, accession numbers GSE72774 and GSE72776.

## Funding

National Institute on Aging (K01AG07204401, F32AG063442), 10.13039/100000066National Institute of Environmental Health Sciences (grant number R21ES032593, 2R01ES010544, R21ES024356, R00ES028743).

## Author contributions

KCP performed statistical analysis. All authors contributed to data interpretation and writing/editing the manuscript. All authors read and approved the final manuscript.

## Declaration of competing interest

The authors declare that they have no known competing financial interests or personal relationships that could have appeared to influence the work reported in this paper.

## Data Availability

The DNA methylation data is available in the GEO repository, accession numbers GSE72774 and GSE72776.
