# Supplementary material for: Immune system disruptions implicated in whole blood epigenome-wide association study of depression among Parkinson's disease patients
Source: Brain Behav Immun Health. 2022 Oct 3;26:100530. doi: 10.1016/j.bbih.2022.100530 (PMC9618774; doi:10.1016/j.bbih.2022.100530)
Supplement: Multimedia component 2 [file mmc2.docx]

**Supplemental Materials**

Depression EWAS CpGs are also related to schizophrenia, aging, and smoking, determined via query of the MRC-IEU catalog of epigenome-wide association studies.

**CpGs related to Schizophrenia**:

EWAS FDR≤0.05: cg23426156, cg11042505

**CpGs related to age:**

EWAS FDR≤0.05: cg18774195, cg26297819, cg09047573, cg16646909, cg01949993, cg21769117, cg13209762, cg24092282, cg06040872, cg09901574, cg09554876, cg23426156, cg21811896, cg22668767, cg15199181, cg06586775, cg05124308, cg05868564, cg22801913, cg24389488, cg21276379, cg07328796, cg08273640, cg19657351, cg00412337, cg11124080, cg01816936, cg07893584, cg11042505, cg13143349, cg08463297, cg25290938

**CpGs related to smoking:**

EWAS FDR≤0.05: cg09047573, cg20476159, cg13209762, cg06040872, cg15199181, cg01816936

**
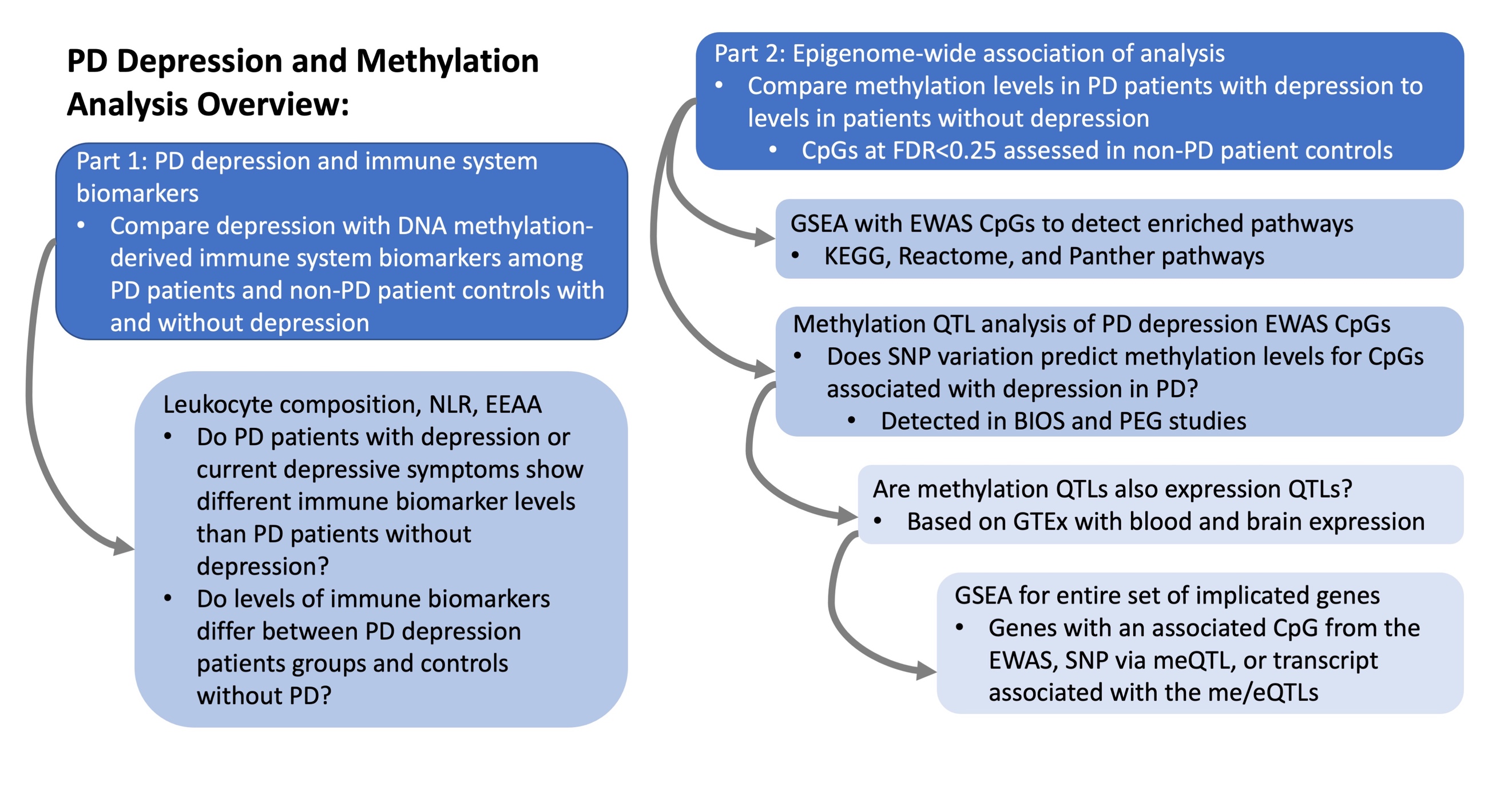
**

**Supplemental Figure 1. Overview of the methylation analysis of PD depression.**


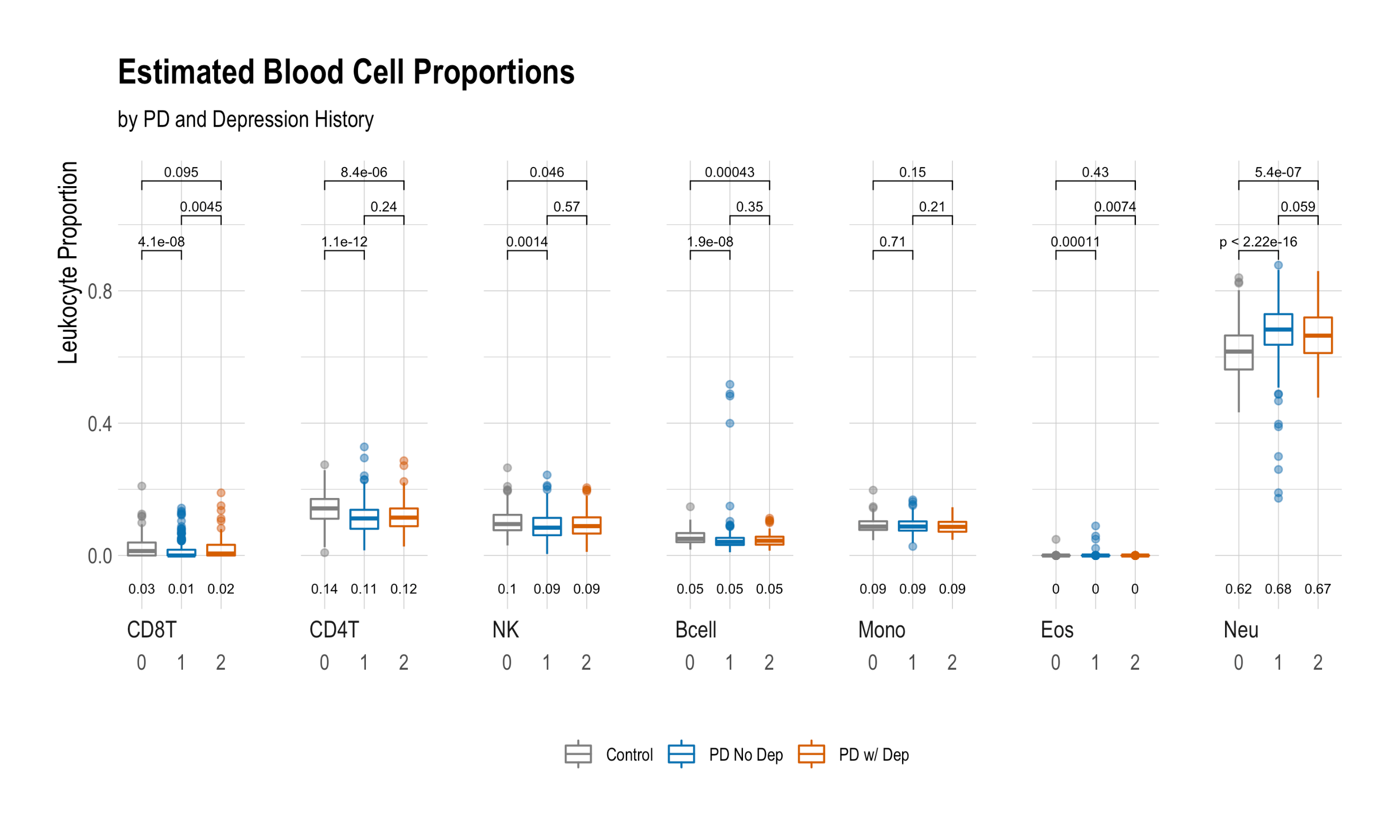


**Supplemental Figure 2. No association between a history of clinical depression and immune cell composition.** PD patients with and without a history of clinical depression show similar leukocyte composition.


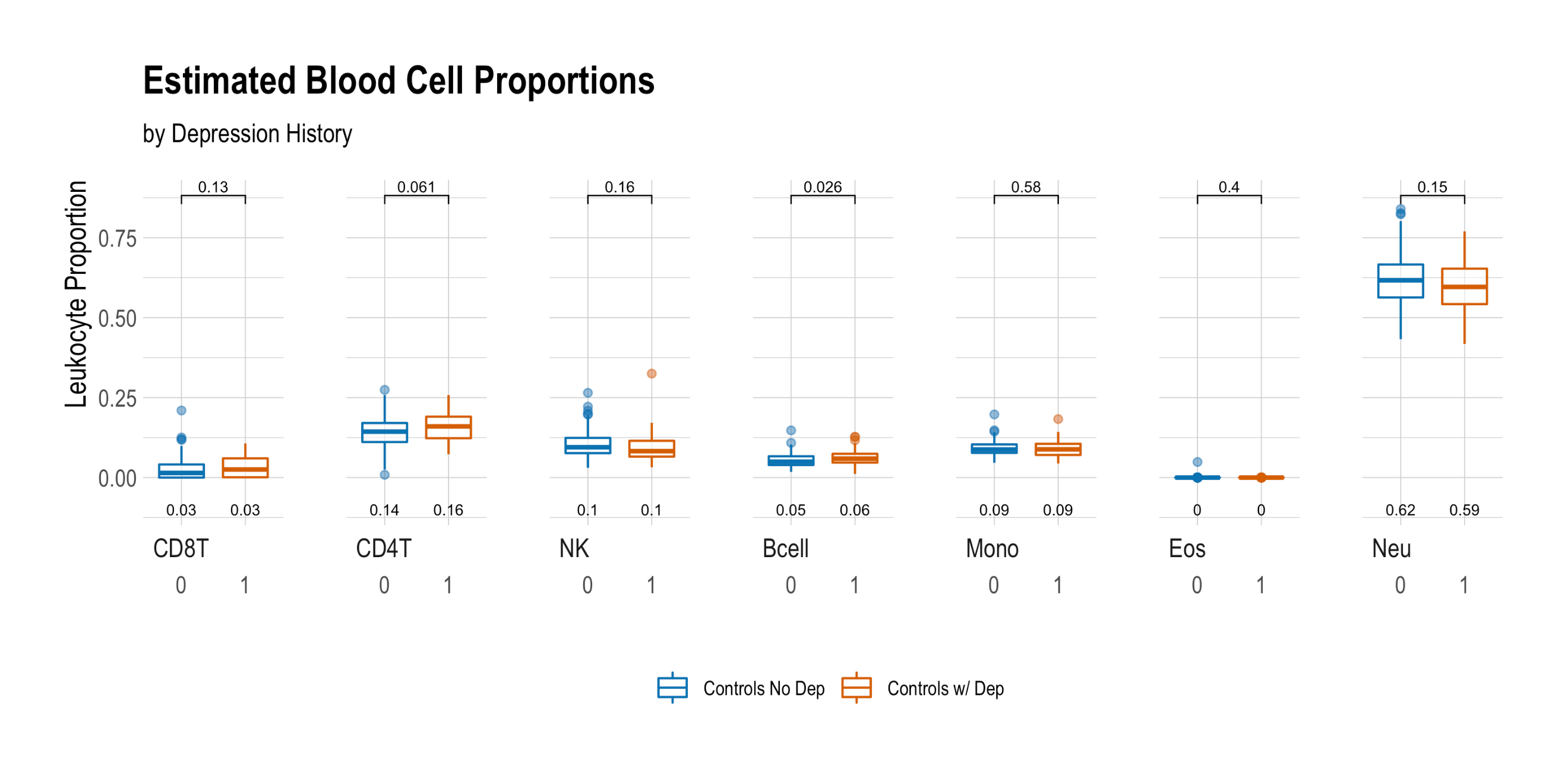


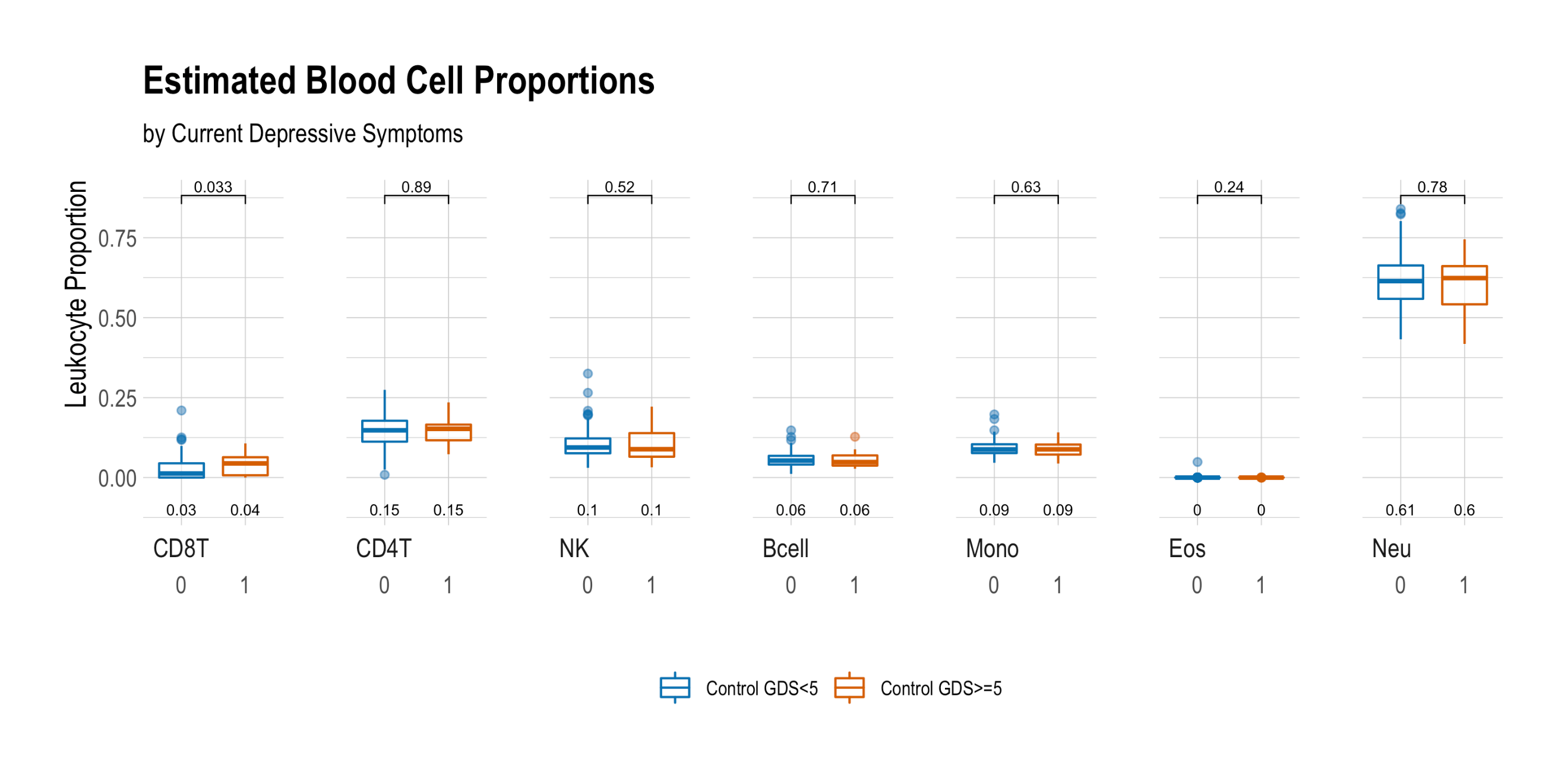


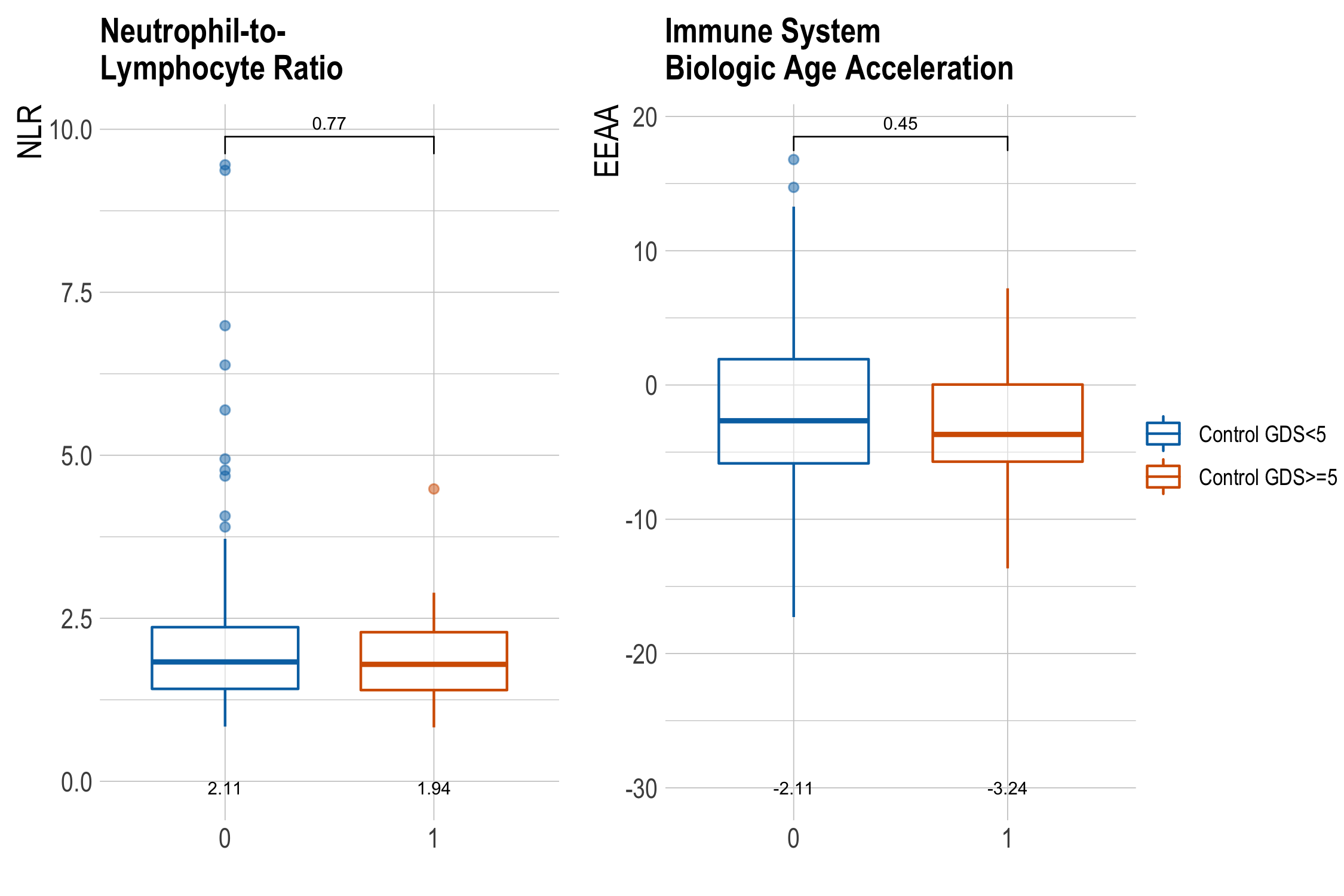


**Supplemental Figure 3. No association between a history of depressive symptoms or clinical depression and immune cell composition among controls.** Controls with and without current depressive symptoms and with and without a history of clinical depression show similar leukocyte composition.

**
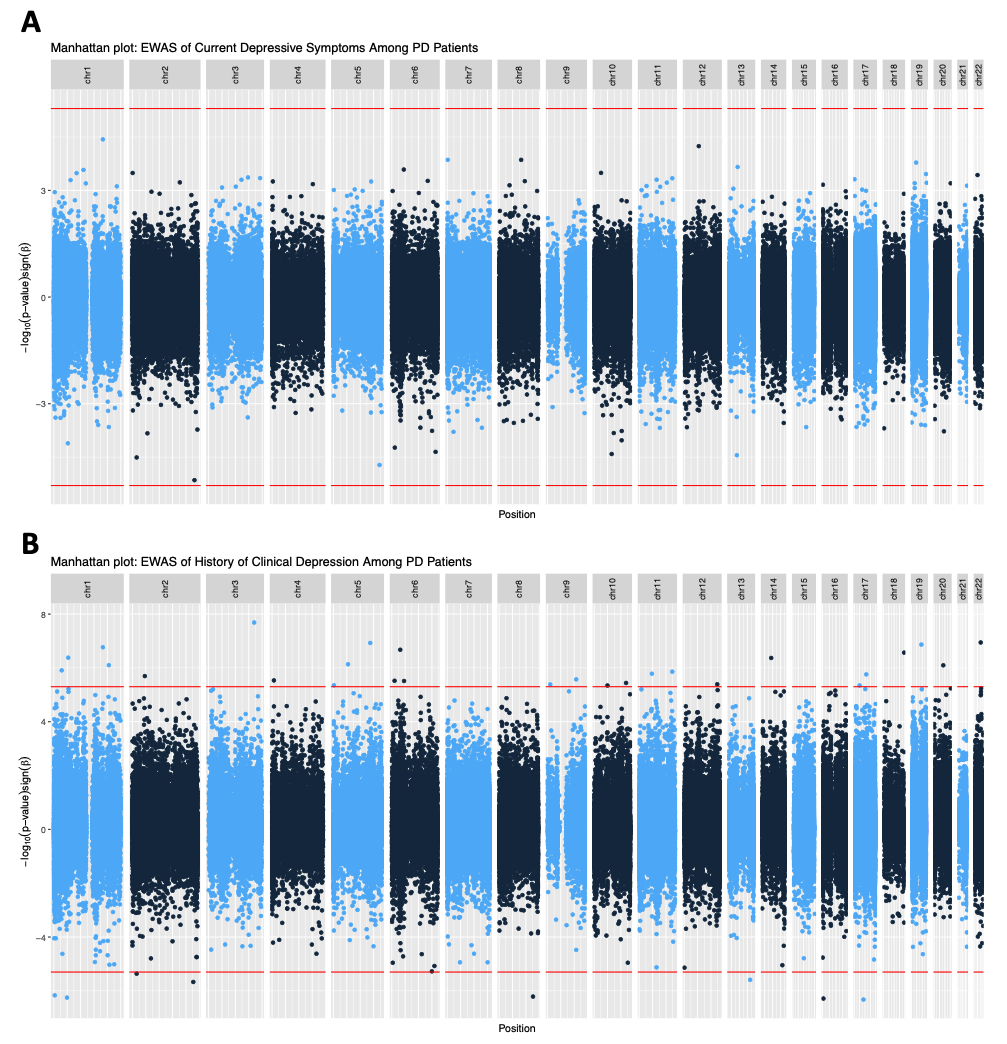
**

**Supplemental Figure 4. Depression and PD EWAS: Differentially Methylation Positions.** Site-by-site CpG EWAS with **A)** current depressive symptoms; and **B)** a history of clinical depression. PD patients of European ancestry only (n=468). EWAS ran via lmFIt with limma (meffil package), controlling for cell composition (CD4T and Neu), age, sex, smoking, PD duration at blood draw, AIMs ancestry (RFvoteCaucasian), and PEG study wave.

**
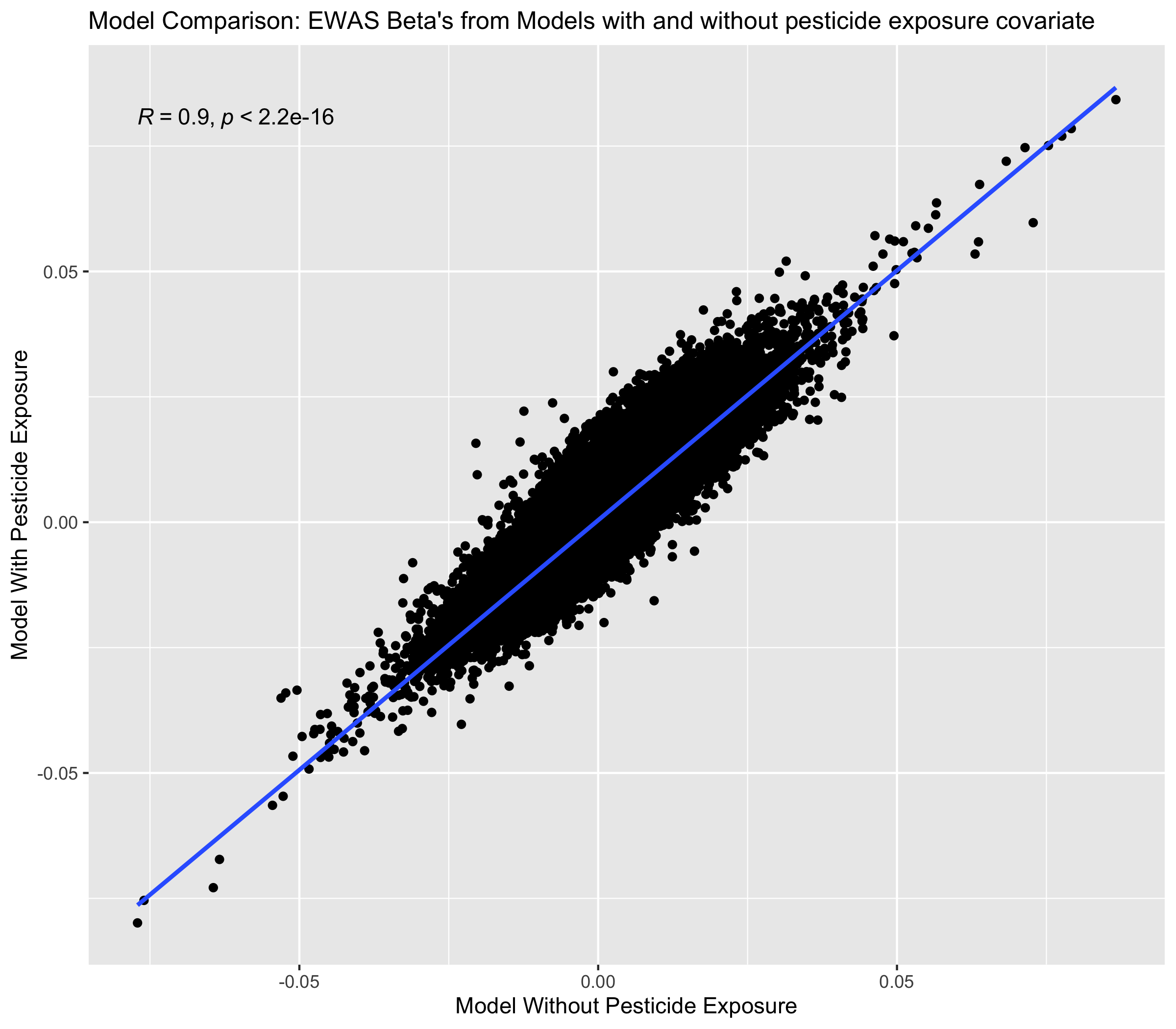
**

**Supplemental Figure 5. Sensitivity Analysis:** comparison of the beta values predicted from EWAS models with and without including pesticide exposure as an additional covariate.

**Supplemental Figure 6.** Mapping cg21769117 (annotated CpG) and it’s three associated me/eQTLs (i.e. SNPs), along with the transcripts the SNPs are associated with, per GTEx expression in whole blood (Top) and also showing all genes in this genetic region (MHC class III), which is very dense gene region (Bottom). This figure demonstrates that the me/eQTLs were not associated with transcript abundance of every gene in the region surrounding cg21769117, but instead selectively associated with the 18 specific transcripts.
